# Cold sensitivity, functional disability and predicting factors after a repaired digital nerve injury

**DOI:** 10.1038/s41598-022-08926-2

**Published:** 2022-03-22

**Authors:** Drifa Frostadottir, Linnéa Ekman, Malin Zimmerman, Stina Andersson, Marianne Arner, Elisabeth Brogren, Lars B. Dahlin

**Affiliations:** 1 Department of Translational Medicine – Hand Surgery, Skåne University Hospital, Lund University, Jan Waldenströms gata 5, 205 02 Malmö, Sweden; 2 Department of Hand Surgery, Skåne University Hospital, Malmö, Sweden; 3 Department of Orthopaedics, Helsingborg Hospital, Helsingborg, Sweden; 4 Department of Hand Surgery, Södersjukhuset, Stockholm, Sweden; 5 Department of Clinical Science and Education, Karolinska Institutet, Södersjukhuset, Stockholm, Sweden

**Keywords:** Outcomes research, Translational research

## Abstract

To investigate self-reported cold sensitivity and functional disability after a repaired digital nerve injury. We identified 3204 individuals operated with digital nerve repair in the Swedish national quality registry for hand surgery (HAKIR). Patient-reported symptoms, including cold sensitivity and perceived disability, were examined using two questionnaires (HQ-8 and QuickDASH), three and 12 months postoperatively. Patients with diabetes (n = 48; 3%) were identified in the Swedish National Diabetes Register (NDR). Cold sensitivity (scored 0–100) was the most prominent symptom among 1553 included individuals (998 men, 555 women; median age 41 [IQR 27–54] years). In the regression analysis, flexor tendon injury, hand fracture and injury to multiple structures predicted worsened cold sensitivity (6.9, 15.5 and 25.0 points; *p* = 0.005, 0.046 and < 0.001) at 12 months. Individuals with moderate (30–70) and severe (> 70) cold sensitivity had higher QuickDASH scores at three and 12 months postoperatively than individuals with mild cold sensitivity (6.0 and 5.5; 19.8 and 21.0 points; *p* = 0.001). Flexor tendon injury, injuries to multiple structures and diabetes had significant effect on QuickDASH scores at three, but not at 12, months postoperatively. Cold sensitivity is common after a digital nerve repair and impacts self-reported disability. A concomitant injury, particularly multiple injuries, predicts postoperative cold sensitivity.

## Introduction

Cold sensitivity can be a severe and debilitating symptom following a variety of hand conditions and injuries, particularly after injuries to the digital and major nerves in the upper extremity^1,2^. Cold sensitivity is defined by four types of symptoms on exposure to cold: (1) pain/discomfort, (2) stiffness, (3) altered sensibility and (4) color change; all of which may occur in isolation or in any combination and severity^3^. Time until symptoms develop varies from immediately up to months after the injury, but in most cases, symptoms appear within the first 6 months and can persist for many years^1,2,4^, with the risk of becoming permanent^5^. Even if cold sensitivity is common after a variety of hand injuries, and particularly after an injury to a peripheral nerve^6^, the phenomenon may be under-reported, partly because most patient-reported outcome measures (PROMs) fail to identify these symptoms and partly because cold sensitivity may be difficult to measure objectively. Specific questionnaires evaluating cold intolerance are available^7–10^, but are too extensive to use on large patient populations. The HAKIR questionnaire (HQ-8;^8,11^) is a newly developed, and validated PROM, which includes questions on seven hand symptoms and one question on hand function, where all questions are scored zero to 100 (0 = no symptoms; 100 = worst outcome). One question regards cold sensitivity, which allows for deeper investigation of the connection of such a symptom to a nerve injury, which to our knowledge has only been related to functional impairment^5,12^ and quality of life^12^, but not been confirmed in a larger population. In this respect, the use of a national quality register represents a novel procedure, where data is continuously collected from patients treated for a digital nerve injury in the health care sector^11^.

A digital nerve injury is often regarded as a minor injury in relation to major nerve trunk injuries or complex hand injuries^13^ and the debate on the need to repair a digital nerve injury is ongoing. Yet, insufficient evidence, based on randomized clinical trials considering also ethical aspects, is available to support alternative methods other than the current practice of carrying out surgical repair^13–15^. However, one should carefully consider the risk of neuroma formation with subsequent pain, where any prevention of neuroma formation is crucial^16^. The outcome after a repaired digital nerve injury and its relevance to sensory re-innervation has recently been highlighted^14,15^. Thus, individuals with poor return of sensation after a nerve injury may have an increased risk of cold sensitivity with related neuropathic pain^17^. Although digital nerve injuries often are associated with concomitant injuries to flexor tendons, digital arteries, or fractures, it is not known whether these associated injuries affect the risk of developing cold sensitivity. Individuals with diabetes and a surgically treated nerve compression lesion, such as carpal tunnel syndrome or ulnar nerve compression at the elbow, seem to be more prone to present with cold sensitivity than individuals without diabetes^18,19^. Thus, evaluation of outcome after a repaired digital nerve injury should not only be related to return of sensation, but also to functional disability and its relation to cold-induced symptoms, as well as to concomitant injuries and co-morbidity, such as diabetes^1,14^.

The main purpose of this study was to investigate the relationship between self-reported cold sensitivity, its associated symptoms and functional disability in a large group of individuals with a repaired digital nerve injury. A second aim was to evaluate how a concomitant injury, isolated or in combination (multiple injuries), and diabetes affect presence of cold sensitivity after a repaired digital nerve injury.

## Results

### Included individuals

**Figure 1:**
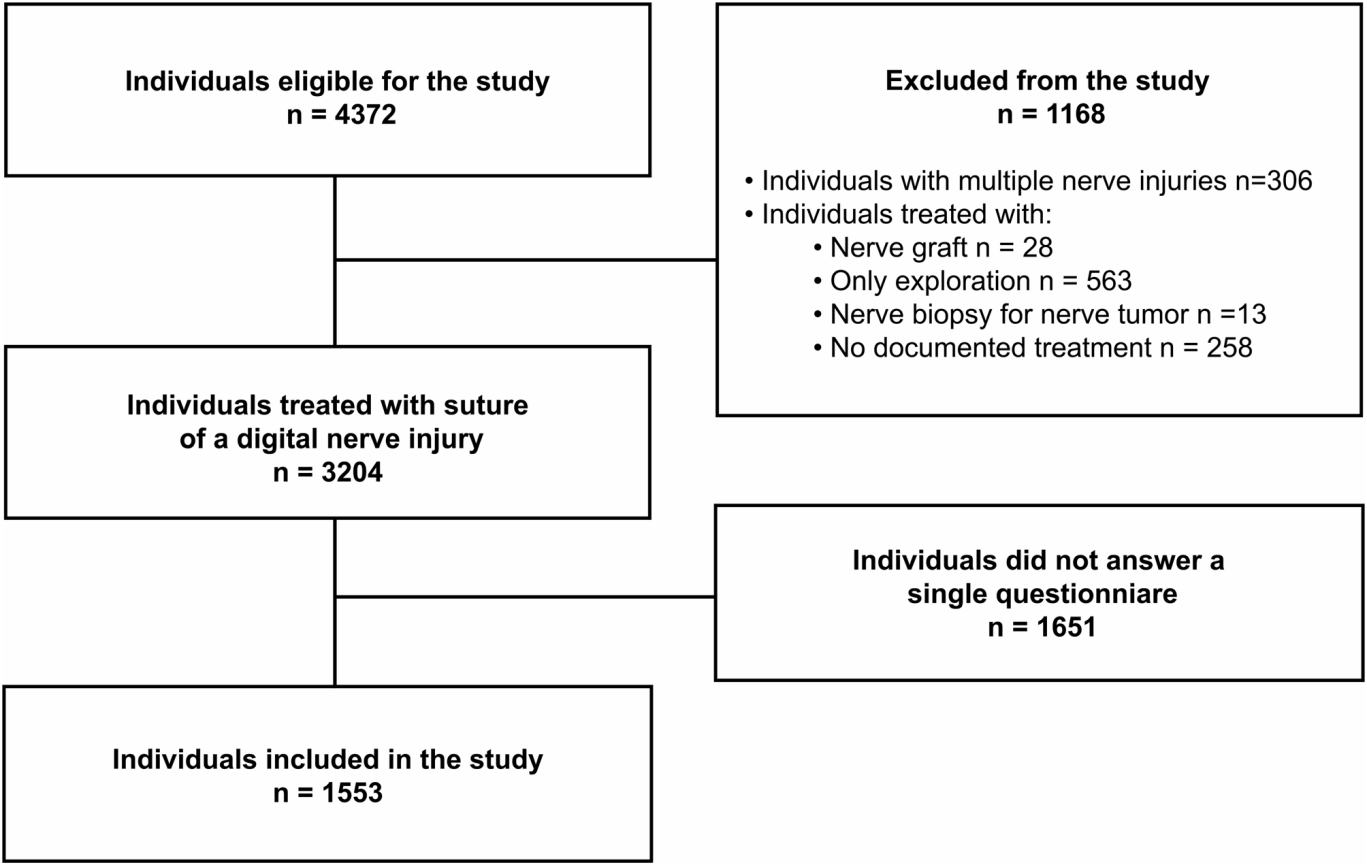
Study patient flow chart.

During the study period, 4372 individuals with a digital nerve injury were identified in the national quality registry for hand surgery (HAKIR). Of those individuals, 28 were treated with reconstruction with a nerve graft, 563 were treated with only exploration, 13 had a nerve biopsy for a nerve tumor, 306 had multiple nerve injuries at hand and wrist (i.e. ICD-10 code S64.7; thus, the code includes both injuries of digital nerves and major nerve trunks), and 258 individuals had no documented treatment for the nerve injury; i.e. all these individuals were excluded. Thus, 3204 individuals remained and were eligible for the study; all of them treated with a direct nerve repair with sutures of a digital nerve with or without concomitant injury in the hand. Of these, 1553/3204 (48%) individuals had responded to at least one HAKIR questionnaire at baseline and/or at 3 months and/or at 12 months postoperatively. Consequently, 1553/3204 (48%) individuals were included in the study (Fig. 1).

### Clinical characteristics

In our study of 1553 individuals, 998 (64%) were men (median age 42 [27–55] years) and 555 (36%) were women (median age 41 [26–51]; *p* = 0.15; Table 1). The left hand was more commonly injured than the right hand (1048/1553, 68%; *p* < 0.001). A diagnosis of diabetes before the nerve injury was confirmed through data obtained from NDR in 48/1553 (3%) individuals (11 type 1 diabetes and 37 type 2 diabetes; Table 1).

**Table 1:** Characteristics and cold sensitivity scores of individuals treated with repair of a digital nerve injury.

	All individuals n = 1553	Men n = 998	Women n = 555
Age at surgery (years)	41 [27–54]	42 [27–55]	41 [26–51]
Diabetes before nerve injury	48 (3)	32 (3)	16 (3)
**Concomitant injuries**			
Flexor tendon injury	557 (36)	386 (39)	171 (31)
Fracture in the hand	28 (2)	26 (3)	2 (0.5)
Joint/ligament injuries	16 (1)	10 (1)	6 (1)
Vascular injury	13 (1)	9 (1)	4 (1)
Multiple injuries	85 (6)	74 (7)	11 (2)
**Cold sensitivity**			
Preoperative cold sensitivity	0 [0–20]	0 [0–10]	0 [0–20]
Cold sensitivity at three months	50 [20–80]	50 [20–72]	50 [20–80]
Cold sensitivity at 12 months	60 [24–80]	60 [30–80]	50 [20–80]
**Seasonal variation**			
Surgery during summer	845 (54)	557 (56)	288 (52)

Values are presented as n (%) or median [IQR; interquartile range]. Cold sensitivity scores from HAKIR questionnaire preoperatively as well as three and 12 months postoperatively.

### Cold sensitivity and related symptoms

None of the symptoms in the HQ-8 questionnaire had completely resolved at 12 months postoperatively, and cold sensitivity was the most prominent self-reported symptom after a repaired digital nerve injury (Table 2). Additionally, no difference in cold sensitivity scores was found between men and women (60 [30–80] and 50 [20–80]; *p* = 0.1) at 12 months postoperatively.

**Table 2:** HAKIR questionnaire 8 (HQ-8) results in 1553 individuals with digital nerve injury treated with suture.

HQ-8 items	Preoperative n = 792	3 months n = 479	12 months n = 760	*p*-value
Cold sensitivity	0 [0–10]^a^	50 [20–77]	60 [25–80]^c^	** < 0.001**
Pain at rest	0 [0–2]^a^	0 [0–10]	0 [0–11]^c^	**0.001**
Pain on motion without load	0 [0–10]^a^	10 [0–27]^b^	10 [0–20]^c^	** < 0.001**
Pain on load	0 [0–30]^a^	28 [10–50]	20 [10–50]^c^	** < 0.001**
Stiffness	0 [0–20]^a^	40 [20–60]^b^	30 [10–60]^c^	** < 0.001**
Weakness	0 [0–17]^a^	31 [10–53]^b^	30 [10–50]^c^	** < 0.001**
Numbness, tingling in fingers	0 [0–30]^a^	40 [10–67]	40 [10–70]^c^	** < 0.001**
Ability to perform daily activities	0 [0–30]^a^	20 [10–43]	20 [1–40]^c^	** < 0.001**

Data are presented as median [IQR; interquartile range]. Significance testing with Kruskal–Wallis and post-hoc Mann–Whitney U-test were used. *Bold values indicate *p*-value < 0.05. *a* indicates *p* < 0.05 between preoperative and 3 months postoperative values; *b* indicates *p* < 0.05 between 3 months postoperative and 12 months postoperative; *c* indicates *p* < 0.05 between preoperative and 12 months postoperative.

*Due to the low number of same responders for all three questionnaires, paired analyses were not applicable for significance testing over time.

### Correlations between Quick DASH, cold sensitivity and related symptoms

There was a moderate correlation (Rho values 0.3–0.7) between cold sensitivity and total QuickDASH score at 12 months (r = 0.598; *p* < 0.001; Table 3). Cold sensitivity also correlated moderately with all other evaluated symptoms in the HQ8, limitations in work and other daily activities (Table 3).

**Table 3:** Correlation between cold sensitivity and other HQ-8 symptoms, n = 786.

	Cold sensitivity (HQ-8)	Pain at rest (HQ-8)	Pain on motion without load (HQ-8)	Pain during loaded exercise (HQ-8)	Stiffness (HQ-8)	Weakness (HQ-8)	Numbness (HQ-8)	Ability to perform daily activities ((HQ-8)	Total QuickDASH score (QD)	Limited in work or other daily activities (QD)
Cold sensitivity (HQ-8)	NA									
Pain at rest (HQ-8)	.394	NA								
Pain on motion without load (HQ-8)	.431	.676	NA							
Pain during loaded exercise (HQ-8)	.506	.538	.717	NA						
Stiffness (HQ-8)	.534	.443	.498	.498	NA					
Weakness (HQ-8)	.549	.460	.558	.640	.691	NA				
Numbness (HQ-8)	.502	.388	.406	.415	.344	.432	NA			
Ability to perform daily activities ((HQ-8)	.552	.499	.571	.637	.565	.697	.459	NA		
Total QuickDASH score (QD)	.598	.565	.669	.712	.573	.718	.548	.767	NA	
Limited in work or other daily activities (QD)	.564	.439	.534	.597	.486	.621	.430	.734	.831	NA

Data are rho-values (Spearman rank test). Only rho-values > 0.3 are presented (i.e. no weak correlations). Rho-values 0.3–0.7: moderate and > 0.7: strong correlation. All *p*-values < 0.001.

### Predictors for cold sensitivity

The multiple regression analysis, including all available variables, showed that a concomitant isolated flexor tendon injury [FPL (flexor pollicis longus); FDP (flexor digitorum profundus) and/or FDS (flexor digitorum superficialis)], a fracture in the hand or injury to multiple structures in the hand predicted higher cold sensitivity scores, evaluated with HQ-8, at 12 months postoperatively with 6.9 points (95% CI 2.0–11.7; *p* = 0.005), 15.5 points (95% CI 0.3–30.7; *p* = 0.046) and 25.0 points (95% CI 15.5–34.5; *p* < 0.001), respectively. Neither age, sex, diabetes, isolated vascular, joint and ligament injuries, nor season at the time of surgery had a significant effect in predicting cold sensitivity at 12 months (Table 4).

**Table 4:** Predicting factors for development of cold sensitivity.

Cold sensitivity	B (95% CI)	*P*-value
Age at surgery (years)	0.017 (− 0.127 to 0.162)	0.81
Sex (male)	− 1.815 (− 6.610 to 2.980)	0.46
Diabetes	− 0.848 (− 12.900 to 11.203)	0.89
Flexor tendon injury	6.894 (2.048 to 11.740)	**0.005**
Fracture in the hand	15.459 (0.260 to 30.659)	**0.046**
Vascular injury	− 6.948 (− 28.424 to 14.529)	0.53
Joint and ligament injury	− 4.251 (− 25.712 to 17.210)	0.70
Multiple injuries	25.012 (15.473 to 34.552)	** < 0.001**
Season (summer)	4.412 (− 0.193 to 9.016)	0.06

Data are presented as B-values (95% confidence intervals). Multiple linear regression with cold sensitivity at 12 months postoperatively as dependent variable. Bold values indicate *p*-value < 0.05.

### Concomitant injuries

The most frequent isolated concomitant injury was a flexor tendon injury (FPL, or FDP and/or FDS), occurring in 557/1553 (36%) of the individuals. Men presented more often with a digital nerve injury in conjunction with an isolated flexor tendon injury than women [386/998 men, (39%); 171/555 women, (31%); *p* < 0.002). Other isolated concomitant injuries were a documented vascular injury at hand or wrist level [13/1553, (0.8%)], fracture in the hand [phalangeal, metacarpal or carpal fracture; 28/1553, (2%)], a joint/ligament injury [16/1553, (1%)], or injuries to multiple structures [85/1553, (6%), i.e. flexor tendon injury [67/85 (79%)], vascular injury [24/85 (28%)], fracture in the hand [59/85 (69%)], and joint/ligament injury [17/85 (20%)]; in combinations] (Table 1). The most common combination of multiple injuries was flexor tendon injury and fracture in the hand, found in [n = 39/85 (46%)] followed by flexor tendon injury and vascular injury [n = 21/85 (25%)].

### Cold sensitivity, QuickDASH, concomitant injuries and diabetes

Individuals with severe cold sensitivity at 12 months postoperatively more often had injuries to multiple structures or a concomitant isolated flexor tendon injury than those with mild cold sensitivity (Table 5). Additionally, these individuals showed higher median QuickDASH scores both at three and 12 months postoperatively compared to individuals with mild and moderate cold sensitivity (Table 5).

**Table 5:** Characteristics and QuickDASH results in cases with mild (cold sensitivity symptom scoring ≤ 30), moderate (cold sensitivity symptom scoring 31–70) and severe (cold sensitivity symptom scoring > 70) cold sensitivity at 12 months postoperatively treated with direct suture of a digital nerve injury [39].

	Mild cold sensitivity n = 195	Moderate cold sensitivity n = 231	Severe cold sensitivity n = 349	*p*-value
Age at surgery (years)	42 [27–57]	43 [30–57]	46 [30–57]	0.44
Sex (men)	109 (56)	148 (64)	225 (65)	0.11
Diabetes before nerve injury	7 (4)	11 (5)	12 (3)	0.70
**Concomitant injuries**				
Flexor tendon injury	64 (33)	88 (38)	147 (42)^c^	0.10
Fracture in the hand	3 (2)	3 (1)	13 (4)	0.12
Joint/ligament injuries	2 (1)	5 (2)	2 (1)	0.21
Vascular injury	3 (2)	3 (1)	3 (1)	0.76
Multiple injuries	3 (2)	11 (5)^b^	36 (10)^c^	** < 0.001**
**QuickDASH score**				
Preoperative QuickDASH	2 [0–39] (n = 59)	5 [0–28] (n = 69)	3 [0–56] (n = 84)	0.34
QuickDASH score at three months	14 [7–20] (n = 73)	14 [9–25] (n = 87)	30 [16–50]^c^ (n = 114)	** < 0.001**
QuickDASH score at 12 months	7 [2–14]^a^ (n = 191)	14 [7–23]^b^ (n = 229)	29 [16–43]^c^ (n = 336)	** < 0.001**
**Seasonal variation**				
Surgery during (summer)	120 (62)	123 (53)	189 (54)	0.17

Values are presented as n (%) or median [IQR; interquartile range]. Bold values indicate *p*-value < 0.05. Significance testing with Kruskal Wallis and post-hoc Mann–Whitney U-test for continuous variables, and Chi Square for nominal variables, where *a* indicates *p* < 0.05 between mild and moderate; *b* indicates *p* < 0.05 between moderate and severe; *c* indicates *p* < 0.05 between mild and severe.

In the univariate general linear regression analysis, moderate [6.0 points (95% CI 2.3–9.7; *p* = 0.002)] and severe [19.8 points (95% CI 16.1–23.4; *p* < 0.001)] cold sensitivity was associated with higher total QuickDASH score at 3 months compared to mild cold sensitivity (adjusted for age, sex, diabetes, isolated flexor tendon injury, isolated fracture in the hand and injury to multiple structures). Furthermore, a significant effect on the total QuickDASH score at the 3 months postoperative follow-up was also shown by isolated flexor tendon injury [4.0 points (95% CI 0.9–7.2; *p* = 0.01)], injury to multiple structures [13.8 points (95% CI 7.3–20.3; *p* < 0.001)], and diabetes [8.6 points (95% CI 1.1–16.1; *p* = 0.02)].

At 12 months, the total QuickDASH score was still higher for moderate [5.5 points (95% CI 2.5–8.4; *p* < 0.001)] and severe [21.0 points (95% CI 18.2–23.8; *p* < 0.001)] cold sensitivity as compared to the group with mild cold sensitivity. None of the adjusting factors (age, sex, diabetes, isolated flexor tendon injury, isolated fracture in the hand and injury to multiple structures), including when adjusting the model for baseline QuickDASH, affected the total QuickDASH score at 12 months (data not shown).

### Responders and non-responders

Of the 1553 individuals included in the study, 837/1553 (54%) responded to the HAKIR questionnaire 8 (HQ-8) preoperatively, 484/1553 (31%) at 3 months, and 772/1553 (50%) responded at 12 months postoperatively. All three questionnaires were completely filled out by 70 responders. When comparing the included individuals to non-responders (i.e. individuals who had not filled in any HAKIR questionnaire at any time-point) the responders were more commonly women than men (55% vs 46%; *p* < 0.001) and were also slightly older (41 [27–54] years) than non-responders (35 [25–49] years; *p* < 0.001).

### Time of surgery

During our study period, 845/1553 (54%) of the surgeries were performed during the summer period (April-September) and 708/1553 (46%) during the winter period (October–March) (Table 1). There was no difference in reported cold sensitivity between individuals operated during the summer period compared to individuals operated during the winter period at three (*p* = 0.53) or at 12 months (*p* = 0.09) postoperatively.

## Discussion

In this large national registry study, cold sensitivity was a prominent self-reported symptom after a repaired digital nerve injury in adults up to 12 months postoperatively. Cold sensitivity was also associated with self-reported disability at three and 12 months after nerve injury repair, as demonstrated by higher QuickDASH scores with a more severe cold sensitivity category. A 20–21 points higher QuickDASH score at three and 12 months after repair of the digital nerve injury in individuals with severe cold sensitivity compared to individuals with mild cold sensitivity is large enough to be clinically relevant^20^. This infers that cold sensitivity may persist in individuals for longer time than 12 months in accordance with data from smaller studies consisting of mainly repaired digital nerves^5,21^. There was no difference in presently reported cold sensitivity symptoms between men and women, nor did sex or age affect the QuickDASH scores at 12 months; the latter in accordance with data from a previous smaller study of cold sensitivity, including different nerve injuries^5^. The present QuickDASH scores for individuals with a digital nerve injury were generally higher (mean score 21.1 at 12 months) than previously reported in smaller retrospective studies of individuals with a digital nerve injury (mean score 7.1–9.0) evaluated at around 30–50 months^22,23^. Our study, as well as the other retrospective studies, included individuals with and without concomitant injuries. However, if multiple digital nerve injuries, also with and without concomitant injuries, are present, the QuickDASH value may approach 40^24^.

The present study of individuals with a repaired digital nerve injury can be considered as representative for a total population^13,21,25^, where men are overrepresented, and the affected individuals are generally young^22^. We only included primary digital nerve repairs, excluding nerve grafts; thus, we present a more homogenous patient population in contrast to previous studies including different surgical methods, such as novel nerve allografts^5,26^, although such surgical procedures all are reported to provide a reasonable outcome^27,28^. Digital nerve injuries often present with concomitant injuries, such as fractures as well as vascular, joint/ligament, flexor tendon and multiple injuries. Presence of a flexor tendon injury, a fracture and multiple injuries increased the severity of cold sensitivity, indicating the complex pathophysiology of development of cold sensitivity, but with the nerve injury considered as the pertinent origin of this symptom. The negative impact of cold sensitivity on activities of daily living decrease over time, but no specific predictor related to a hand injury can be identified as relevant for such a reduced cold sensitivity^29^. However, the nerve injury itself, and not the repair or the reconstruction of the injury, is probably the most relevant factor for development of cold sensitivity; a statement in accordance with the concept of the severe cold sensitivity, with or without white fingers, in Hand Arm Vibration Syndrome (HAVS) in which mainly a nerve injury is present^29,30^. Concomitant injuries were more common in men, indicating a more severe injury. This may reflect the fact that men more commonly suffer from work related injuries^31^, although work-related injuries among the working population is low in Sweden^32^. Social division of labor, where men take on heavy and dangerous tasks^33^, may also play a role in that men suffer from more extensive injuries to the upper limb, which seems to be true also in an elderly population^34^. In our material, a flexor tendon injury was by far the most common isolated concomitant injury and more often seen in individuals with severe cold sensitivity at 12 months postoperatively than in those with mild cold sensitivity. Such an injury had a significant impact on arm-related disability at three, but not at 12 months. This corresponds well with the recovery time after a surgically repaired flexor tendon injury, in which full strength of the tendon first is achieved approximately 3 months postoperatively, when the individual is allowed to put full load on the injured hand. In addition, a digital nerve injury does not affect postoperative range of motion after a flexor tendon injury^35^; an important aspect when evaluating combined injuries. Altogether, this indicates that the prolonged disability after a repaired digital nerve injury at 12 months can be related to presence of cold sensitivity.

Based on previous published studies, data indicates that the most relevant factor in developing cold sensitivity is probably the nerve injury itself, since the most severe symptoms develop after such an injury^4,29^. Yet, injuries to multiple structures in the hand were associated with more severe cold sensitivity at 12 months postoperatively. This may be explained by a more extensive injury and disability; again, highlighting the relevance of evaluating outcome in combined injuries^7,36^. The individual may be battling not only the symptoms from the digital nerve injury, but also the healing process of a fracture and/or flexor tendon injury with associated stiffness and weakness, which is presently shown to be associated with increased cold sensitivity. A phalangeal fracture associated with a digital nerve injury has previously been reported not to further impair function measured as range of motion^36^; however, the authors did not evaluate any detailed nerve function after such fractures with a digital nerve injury^36^. Attention is also called to the fact that a vascular injury, isolated or in conjunction with other concomitant injuries, was reported in very few cases (3%). When considering the proximity to the digital nerves, this was a surprising finding. We speculate that only the cases needing a vascular repair were reported in the register. Most often, the arterial supply of the finger is not at risk when a single digital artery is injured and is therefore not always repaired with microsutures. Still, in accordance with the concept of cold sensitivity in HAVS, it is the digital nerve injury per se, and not the fact that it is repaired or reconstructed, that is the primary cause of the cold sensitivity. Nerve regeneration and functional recovery after any nerve injury in humans is not complete even after surgical treatment^37^. In addition, a nerve compression lesion, such as carpal tunnel syndrome, induces cold sensitivity, which disappears after surgery with decompression of the nerve. Altogether, this indicates that it is the nerve injury that is causing symptoms such as cold sensitivity^18,38,39^.

There was no difference in cold sensitivity between individuals operated during the winter period compared to those operated during the summer period. The individuals in our study were treated at hospitals and units along Sweden; thus, with different climate during the various time periods. Previous studies on cold sensitivity and its severity support this finding, showing that cold sensitivity results are very similar between regions with different climate and temperatures^9,10^, although a seasonal variation in cold sensitivity has been reported after repair of various nerve injuries in a smaller study^5^.

Previous studies have reported occurrence of cold sensitivity after a peripheral nerve injury, as well as in individuals with other nerve conditions, such as carpal tunnel syndrome, and its association with pain on motion without load, pain on load, stiffness, weakness and numbness/tingling^39^. These symptoms correlated moderately to each other, as well as with activities and disability, and are debilitating symptoms that should be considered when deciding on treatment for nerve injuries^1,17,39^. In the present study, individuals with severe cold sensitivity scored high on all other HQ-8 items. Thus, cold sensitivity seems to be a prominent feature in perceived disability after a repaired digital nerve injury. This prolonged symptom can affect return to work, especially certain types of professions, where work in cold and damp environments, including construction work during winter or working with frozen goods, can be extremely painful, as well as prohibiting individuals from pursuing leisure activities as skiing and other winter sports. This should be considered in view of the ongoing debate whether repair of even a single digital nerve is necessary. It has been claimed that, since regain of normal sensation, i.e. 2-PD, is unlikely at least among adults, a digital nerve repair is not required, but more knowledge is needed to fully support such a statement^14,15^. However, if a sensory nerve, such as a digital nerve in the hand or the superficial branch of the radial nerve in the forearm, is not, or insufficiently, repaired, there is a substantial risk of neuroma formation with subsequent pain^16^. Such neuroma can be treated either by a nerve transposition (i.e. passive approach) or by a nerve repair or reconstruction (active approach)^40^ with improvement of pain, but still with a risk of reoperation(s) and residual pain^16^. An active approach may be a preferable procedure in such a situation^41^. Thus, such data indirectly indicates that a digital nerve injury still should be repaired or reconstructed in clinical practice. Although cold sensitivity and neuroma are known complications after nerve injuries, most studies on outcome after nerve repair focus on the return of sensibility, in particular recovery of 2-PD^14^. Considering our findings of individuals with a digital nerve injury reporting substantial cold sensitivity and related symptoms as well as that cold sensitivity is associated with higher arm-related disability, we suggest that cold sensitivity is an important outcome that should be evaluated in addition to sensibility in individuals with a repaired digital nerve injury.

The proportion of individuals with diabetes in our study, consisting of around 3%, represents the prevalence of diabetes in Sweden (30), but is reported to be higher in population studies with nerve compression lesions, such as carpal tunnel syndrome and ulnar nerve compression at the elbow (around 12%), indicating an increased susceptibility to nerve compression. In view of the number of subjects with diabetes having an active professional and leisure life due to the development of the care of the diabetes with meticulous monitoring of blood glucose and thereby being active at the labor market, there may be an equal risk for hand injuries, and particularly nerve injuries, among such individuals as in individuals without diabetes. Thus, it is important to also report outcome in individuals with diabetes. As for the isolated concomitant flexor tendon injury, individuals with diabetes had a higher QuickDASH score at three, but not at 12, months postoperatively, indicating a transient, but not permanent, increased disability. This can be explained by the fact that nerve regeneration, both of the large myelinated and the small non-myelinated nerve fibers, may be slower in subjects with diabetes^42,43^.

Due to this being a register study, where information is collected via the HQ-8 questionnaire and QuickDASH, there were limitations in the material. We had no knowledge of previous injuries, as well as the general health of the individuals and their habits, e.g. smoking^21,44^, rheumatic disease and body mass index (BMI), which may directly or inversely influence the presence of cold sensitivity^45,46^. The HQ-8 questionnaire is a much simpler questionnaire than the CISS^47^, which plays a role in the limitations of material, while also making it easier to engage more individuals to partake. The relevant ICHOM (International Consortium for Health outcomes Measurements) recommendations for regular and extended evaluation of outcome after nerve injuries include several components, including questionnaires, and require more time in clinical practice; thus, being more time consuming^48^. There is also the possibility that symptomatic subjects might be more prone to respond to a questionnaire of this kind, which may lead to an overestimation, rather than an underestimation, of symptoms, such as cold sensitivity. In accordance with other register studies, women responded more often to the questionnaires than men and responders were also older than non-responders, indicating that the present data are comparable with other studies and valid^39,49^. Another limitation was the low number of same responders to all three questionnaires. Thus, paired analyses were not applicable for significance testing over time. On the other hand, the strength of our study is the large study population, being one of the largest presented studies, which allowed for a more generalized approach. Finally, we have no information about the use of loupes or operating microscope during the surgery, but that most probably does not affect the outcome^23,44^. The strength of the present study is the well-defined inclusion of a repaired digital nerve injury, with and without a concomitant injury, including multiple injuries, in contrast to some previous studies^50^, although it has been claimed that for example various surgical methods may provide similar results^28^.

## Conclusion

We conclude that cold sensitivity after a repaired digital nerve injury is a common self-reported symptom, and is associated with worse self-reported disability. Cold sensitivity needs to be considered when evaluating outcome after treatment of digital nerve injuries with and without, isolated or multiple, concomitant injuries and its relation to comorbidity.

## Methods

### Study design, participants and data sources

The Swedish national quality registry for hand surgery (HAKIR) collects data from performed hand surgical procedures at seven university hospitals and presently also at four private units in Sweden^11^. In this retrospective study, individuals aged 16 or above, operated with surgical repair of a digital nerve injury 2010–2018, were eligible for the study. Individuals were identified through ICD10 diagnosis codes (International Statistical Classification of Diseases and Related Health Problems^51^) S644 and S643 and surgical procedure code (KKÅ97) ACB29. The exclusion criteria were surgery with nerve graft, nerve biopsy and combined injuries to both digital nerves and nerve trunks. The individuals provide informed consent prior to inclusion in the registry and are asked to fill in the HAKIR Questionnaire-8 (HQ-8) and QuickDASH at baseline (preoperatively) and at three and 12 months postoperatively, either by post or online. The HQ-8 questionnaire consists of eight validated Likert-scale questions assessing symptoms (i.e. pain on load, pain on motion without load, pain at rest, stiffness, weakness, numbness/tingling, cold sensitivity and ability to perform daily activities^8^). Each symptom is graded by the individual from 0 to 100, with a higher score representing more severe problems^8^. The HQ-8 questionnaire is not a disease-specific outcome instrument, but it complements other PROMs, such as the QuickDASH questionnaire, with symptom-related questions and also shares some features with the validated Cold Intolerance Symptom Scale (CISS). Yet, the HQ-8 questionnaire is considered somewhat simpler, making it easier to use in routine clinical practice and to engage a larger population to partake^9,47^. Furthermore, when the national quality register HAKIR was started in 2010, the ICHOM recommendations for regular and extended evaluation of outcome after nerve injuries were not available^48^. We classified individuals into three categories according to the severity of cold severity agreeing to a previously published study^39^. Mild cold sensitivity was defined as a HQ-8 score of ≤ 30, moderate cold severity as a score of 30–70 and severe cold severity as a score of > 70. The validated Swedish version of the QuickDASH questionnaire assesses self-reported arm-function and is scored from 0 to 100, where a higher score represents more severe disability^52^. Data on any isolated and multiple concomitant injuries, such as a flexor tendon injury, fracture in the hand (i.e. phalangeal, metacarpal or carpal fracture), a joint/ligament injury, a vascular injury (at hand or wrist level) or multiple injuries, was also retrieved from the HAKIR database. Injury to multiple structures included flexor tendon injury, fracture in the hand, vascular injury and/or joint/ligament injury in various combinations (Fig. 1).

The Swedish National Diabetes Register (NDR) is a national quality register that includes all individuals above 18 years diagnosed with type 1 or 2 diabetes (http://www.ndr.se). Each individual provides informed consent before inclusion in the register. The HAKIR database was linked to NDR through personal identifying numbers and data on the individuals’ diabetic status in our study was retrieved.

### Ethical approval and consent to participate

This study was approved by the Regional Ethical Review Board in Stockholm, Sweden, and the national Ethical Review Board (2017/3:11 and 2021–00,902). The research was performed according to the Helsinki Declaration.

### Statistics

Data are presented as median [interquartile range, IQR] or numbers (%). The Chi-Square test was used for nominal variables when comparing individuals with or without a concomitant injury as well as comparing individuals evaluated and categorized as mild (HQ-8 score of ≤ 30), moderate (HQ-8 score of 30–70) and severe (HQ-8 score of > 70) cold sensitivity at 12 months. Fisher´s exact test was used to compare the frequency of the operated hand (left vs right hand). The Kruskal–Wallis test was used for significance testing for continuous variables, with Mann–Whitney U-test as the post-hoc test. Mann–Whitney U-test was also used for sex, age and seasonal comparisons. In order to predict cold sensitivity outcome, a multiple linear regression analysis was performed with cold sensitivity at 12 months as the dependent continuous variable, adjusted for all available factors as age, sex, diabetes, isolated flexor tendon injury, isolated fracture in the hand, isolated vascular injury, isolated joint/ligament injury, injuries to multiple structures (i.e. flexor tendon injury, fracture in the hand, vascular injury and/or joint/ligament injury in various combinations) as well as season for surgery. To assess the association between cold sensitivity and functional disability, a univariate general linear analysis was performed with the QuickDASH score at 12 months as the dependent continuous variable and the three cold sensitivity categories as the independent variable. The model was adjusted for age at surgery, sex, diabetes, baseline (preoperative) QuickDASH score, as well as isolated flexor tendon injury, isolated fracture in the hand and injury to multiple structures that showed be predicting factors for cold sensitivity outcome in the multiple regression analysis. Other concomitant isolated injuries were not selected as adjusting factor due to the low numbers of occurrence as well as not being predicting factors to cold sensitivity outcome in the multiple regression analysis. Values are expressed in points as unstandardized B values with 95% confidence intervals. Spearman rank test was used for correlation testing of cold sensitivity and remaining HQ-8 questions and QuickDASH, presenting only moderate (0.3–0.7) and strong (> 0.7) rho values; i.e. excluding weak correlations. A *p*-value of < 0.05 was considered statistically significant, unless otherwise stated.

## Data Availability

The datasets generated and/or analyzed during the current study are not publicly available. Public access to data is restricted by the Swedish Authorities (Public Access to Information and Secrecy Act; https://www.government.se/information-material/2009/09/public-access-to-information-and-secrecy-act/), but data can be available for researchers after a special review that includes approval of the research project by both an Ethics Committee at the national level (www.etikprovningsmyndigheten.se) and the authorities’ data safety committees (such as “KVB-decision”).
